# How does digital inclusive finance enhance rural economic resilience? — A study based on provincial panel data in China

**DOI:** 10.1371/journal.pone.0321630

**Published:** 2025-04-09

**Authors:** Yunlong Ma, Ruolan Wei, Huina Bi

**Affiliations:** 1 Department of Business Administration, Kyungil University, Gyeongsan-si, Republic of Korea; 2 Department of Humanities and Social Sciences, Hebei University of Environmental Engineering, Qinhuangdao, Hebei, China; Thammasat University, THAILAND

## Abstract

This paper explores the role of digital inclusive finance in enhancing rural economic resilience, using panel data from 30 Chinese provinces, municipalities, and autonomous regions from 2013 to 2023. The results show that digital inclusive finance significantly improves rural economic adaptability and transformation capabilities, though its impact on risk resistance is minimal. It strengthens rural economic resilience through two main channels: improving transportation infrastructure and promoting rural technological innovation. This effect is particularly strong in areas with advanced rural digital infrastructure. Heterogeneity analysis reveals that digital inclusive finance positively impacts rural economic resilience in both eastern and western regions, but has no significant effect in central regions. Furthermore, its impact is more pronounced in non-agricultural provinces compared to agricultural ones. The study suggests that the government should continue expanding digital inclusive finance, while tailoring policies to local conditions, to support the sustainable development of the rural economy.

## Introduction

Addressing the “Three Rural Issues”—rural areas, agriculture, and farmers—is fundamental to ensuring national welfare and tackling the country’s most pressing livelihood challenges. At the 2023 Central Rural Work Conference, General Secretary Xi Jinping provided important directives on “Three Rural Issues,” emphasizing the need to “prioritize the development of agriculture and rural areas... and accelerate the modernization of agriculture and rural areas to better advance Chinese-style modernization.” In the same year, the Central Document No. 1 underscored the significance of “resilience” in building a robust agricultural nation and called for enhancing rural governance to cultivate more resilient and competitive agricultural supply chains.

The concept of resilience was first introduced in 1973 by scholar Holling, who used it to assess the persistence and recovery capacity of ecosystems following shocks [1]. Later, the concept was extended to the field of economics to explain the varying abilities of different economic entities to adapt to and recover from economic challenges. The unpredictability of the sources, scope, and intensity of shocks has long posed a challenge for governments worldwide. The inevitability of certain shocks and their profound, lasting impacts have driven countries to focus on enhancing the endogenous capacities of their economies—specifically, by strengthening their ability to resist and adapt to external shocks, thereby enabling rapid economic recovery. This, at its core, represents the essence of the concept of “resilience.”

According to data from 2024, the added value of China’s primary industry accounted for 6.8% of its GDP, and the steady, positive trend in the agricultural and rural sectors has remained unchanged. However, the rural economy continues to face multiple risks, such as natural disasters, market fluctuations, and technological innovations. Notably, in recent years, the aging of the labor force and the structural contradictions in food supply have become increasingly prominent, further exacerbating the volatility and vulnerability of agricultural systems [2]. In this context, enhancing the resilience of the rural economy has become a key driver for promoting high-quality rural development. Rural economic resilience refers to the ability of the rural economy to resist risks, adapt, and recover when faced with external shocks (such as natural disasters or economic crises). It not only represents a response to existing economic conditions but also emphasizes the capacity of the rural economy to rapidly recover after a shock and achieve sustainable development through long-term innovation and adjustments [3].

Given the significant role of rural economic resilience, enhancing this resilience has become a central focus of research. This study examines methods for improving rural economic resilience from the perspective of digital inclusive finance. Digital inclusive finance is an innovation and extension of traditional inclusive finance. The primary objective of inclusive finance is to address issues such as wealth inequality and the imbalanced allocation of financial resources, and it plays a vital role in supporting China’s comprehensive rural revitalization. Traditional financial services often exhibit a “rich-preference” bias, especially in rural areas where factors such as poor credit environments, lack of collateral, fragmented living patterns, and income instability frequently prevent rural residents and small and micro enterprises from accessing sufficient financial services [4,5]. In 2005, the concept of inclusive finance was first introduced during the United Nations’ “International Year of Microcredit” and was formally defined in the 2006 UN blue paper, *Building Inclusive Financial Systems*. Inclusive finance is defined as providing appropriate financial services to all social strata, particularly vulnerable groups such as small and micro enterprises, farmers, and low-income urban populations, at affordable costs. In 2015, China’s State Council issued the *Plan for Promoting the Development of Inclusive Finance* (2016–2020), further advancing the development of inclusive finance in China. The goal of inclusive finance is to offer broader and more convenient financial services to all individuals and enterprises in need of financial support.

With the arrival of the digital information age, the concept of digital inclusive finance was formally introduced at the 2016 G20 Hangzhou Summit. Digital inclusive finance refers to the use of digital technologies, such as mobile payments, electronic wallets, and big data, to promote the development of inclusive finance, particularly in rural areas and for small and micro enterprises. In China, the implementation of digital inclusive finance is multifaceted, including fintech companies providing convenient payment services to rural residents through platforms such as Alipay and WeChat Pay, utilizing big data to assess farmers’ creditworthiness and offer loans and insurance, and enhancing the transparency and security of rural finance through blockchain technology. Additionally, some regions have established government-led digital financial service platforms to offer financing support to local small and micro enterprises [6]. By combining the advantages of “digital technology” and “inclusive finance,” digital inclusive finance extends financial services into areas traditionally underserved by conventional financial systems, significantly increasing coverage for the “Three Rural Issues” and improving access to financial support for vulnerable groups [7]. Digital inclusive finance has played a transformative role in reshaping the rural financial ecosystem and driving high-quality growth in the rural economy.

According to the *China Inclusive Finance Development Report (2022)*, by the end of 2022, mobile payment users in rural areas exceeded 400 million, while the balance of internet loans surpassed 1 trillion yuan. The rapid development of digital inclusive finance in rural areas has garnered significant academic attention, with scholars exploring its relationship with rural economic resilience. However, studies directly addressing this specific topic remain relatively limited. This paper systematically reviews and analyzes relevant literature from two perspectives. The first perspective examines the impact of digital inclusive finance on economic resilience. Recent studies have investigated how digital inclusive finance enhances economic resilience at both provincial and city levels, yielding consistent findings. Research based on provincial samples demonstrates that digital finance positively contributes to resilience, resistance, adaptability, and the transformative capacity of regional economies [8,9]. Moreover, the development of digital finance shows a marginally increasing effect on the economic resilience of both regional and neighboring provinces [10]. At the city level, scholars have found that digital inclusive finance significantly strengthens urban economic resilience by enhancing innovation capacity, boosting consumer spending, and promoting coordinated green innovation [11]. Additionally, some studies suggest a nonlinear relationship, where the positive effects of digital finance on urban economic resilience increase with improvements in industrial structure and local government attention [12].

At the industrial level, research indicates that industrial diversity strengthens economic resilience by reducing the risk of cascading failures that occur when an economy depends on a single industry during economic shocks [13]. Upgrading the industrial structure further strengthens resilience by facilitating the transfer of production factors, optimizing resource allocation, and boosting the economy’s capacity to withstand market risks [14]. Additionally, higher levels of industrial agglomeration not only enhance local economic resilience but also create external economies of scale, reducing risks and costs within urban clusters and generating spatial spillover effects that promote resilience in neighboring areas [15]. At the policy level, studies suggest that reducing policy uncertainty [16], ensuring regional policy coordination [17], and enabling timely responses from policymakers [18] are key to improving economic resilience. In terms of technological innovation, scholars argue that technological advancements, particularly in information and communication technologies, artificial intelligence, and digital technologies, significantly enhance regional economic resilience [19]. Research also shows that the development of transportation infrastructure, like light rail and high-speed rail, positively impacts economic resilience [20]. Areas with better transportation accessibility experience higher employment densities and greater economic dynamism, while infrastructure development in central cities significantly boosts regional resilience, with diminishing effects on peripheral areas [21].

While previous studies have emphasized the role of digital inclusive finance in boosting regional and urban economic resilience, challenges like poor infrastructure and limited financial literacy prevent it from being fully effective in rural areas. Thus, questions remain: Does digital inclusive finance enhance economic resilience in rural areas? What mechanisms drive this effect, and how can barriers be overcome? These questions require further empirical investigation.

This paper addresses these gaps by exploring the relationship between digital inclusive finance and rural economic resilience. It introduces several innovations: First, while much of the research focuses on urban or regional resilience, this study specifically examines rural economic resilience, expanding the understanding of digital inclusive finance in rural contexts. Second, the paper identifies and confirms how digital inclusive finance enhances rural economic resilience, mainly by improving transportation infrastructure and promoting technological innovation, while highlighting the moderating role of rural digital infrastructure development. This approach not only broadens the research scope but also offers new insights into the application of digital inclusive finance in rural economies.

## Theoretical analysis and research hypotheses

### Direct impact effect

Digital inclusive finance is a modern financial model that integrates digital technology with financial services. For financial institutions, it offers two key advantages. First, it reduces reliance on physical branches, thereby lowering operational and infrastructure costs, shortening service delivery times, and enhancing overall efficiency [22]. Second, it mitigates default risks arising from information asymmetry. Prior to lending, financial institutions can assess the creditworthiness of farmers using big data, which helps address adverse selection issues. After the loan is granted, financial institutions can monitor fund usage and evaluate credit ratings, thereby minimizing moral hazard. This reduces the credit constraints commonly found in traditional financial systems and increases credit availability in rural areas.

For rural loan recipients, digital inclusive finance extends financial services to remote areas, increasing service points, expanding coverage, and lowering entry barriers. This improves access to equitable and affordable financial services, alleviates funding constraints, reduces high loan costs in large-scale production, and diversifies funding sources for scaled operations. By promoting the “mass entrepreneurship and innovation” strategy in rural areas, digital inclusive finance not only creates employment opportunities for farmers [23] and boosts income but also fosters industrial diversification, stimulates technological innovation [24], enhances risk resistance, and strengthens rural economic resilience.

The impact of digital inclusive finance on rural economic resilience also exhibits heterogeneity. Variations in the development levels of different dimensions of digital inclusive finance lead to differing effects [25]. For instance, broader coverage and deeper usage address the spatial and temporal limitations of traditional finance, while higher digitalization levels improve ease of access [26]. Additionally, regional economic disparities influence the overall development of digital inclusive finance. Economically advanced eastern regions offer funding and technical support, while western and central regions, despite rapid growth under the “Western Development Strategy” and “Central Rising Strategy,” still lag behind. Development levels generally follow the order: eastern> central> western. These regional differences, combined with the scale of agricultural production, result in varying degrees of economic resilience across regions.

H1: Digital inclusive finance enhances rural economic resilience, and its impact varies across regions due to heterogeneity in development levels and agricultural production scales.

### Mediating effect

Existing literature has explored the roles of financial service quality, information-sharing mechanisms, education levels, transportation infrastructure, and technological innovation as potential mediating variables in the relationship between digital inclusive finance and rural economic resilience [27]. However, empirical tests quantifying these transmission mechanisms remain limited. Building on previous research, this study argues that digital inclusive finance can directly improve the accessibility of financial resources in rural areas, which, in turn, influences investments in transportation infrastructure and technological innovation—both of which are critical drivers of rural economic transformation. As such, this study selects transportation infrastructure and technological innovation as mediating variables for a more focused analysis of the underlying mechanisms. While other factors may also affect rural economic resilience, their complex and indirect relationships with digital inclusive finance suggest that they operate through multiple intermediary pathways. To simplify the model and maintain a clear research focus, these complex factors are treated as control variables. The subsequent sections will explore in greater depth the mediating mechanisms of transportation infrastructure and technological innovation.

Transportation infrastructure plays a critical role in rural economic development. It not only directly influences the quality of rural economic growth but also serves as a key mediating factor in how digital inclusive finance impacts rural economic resilience. First, digital inclusive finance supports the development of transportation infrastructure by increasing the availability and accessibility of financial resources in rural areas [28]. It facilitates infrastructure construction by providing convenient loans and financing channels, enabling local governments and businesses to access the necessary funds for development projects. Second, improved transportation infrastructure enhances production efficiency and market connectivity in rural economies. A well-developed transportation network reduces logistics costs and increases the efficiency of agricultural product transportation, enabling rural production materials and goods to reach markets more quickly. This improvement not only increases the frequency and profitability of rural economic activities but also enhances the economy’s ability to withstand external shocks, like market fluctuations and natural disasters. Additionally, high-quality transportation infrastructure attracts external investment, fostering industrial upgrades and further enhancing rural economic resilience. Finally, digital inclusive finance indirectly strengthens rural economic resilience through its impact on transportation infrastructure. By accelerating infrastructure development, the financial services provided by digital inclusive finance enhance rural economies’ risk resistance and adaptability. Furthermore, improved transportation infrastructure amplifies the effectiveness of digital inclusive finance, allowing financial services to reach rural areas more efficiently and widely, thereby further reinforcing economic resilience.

H2: Digital inclusive finance enhances rural economic resilience by improving transportation infrastructure.

Technological innovation is a key driver of economic development and plays a crucial role in enhancing economic resilience [29]. It improves agricultural productivity, optimizes industrial structures, and strengthens risk resistance. Digital inclusive finance fosters rural technological innovation by providing convenient and efficient financial services. Using advanced technologies like big data and artificial intelligence, digital inclusive finance offers targeted financial support to innovation-driven entities in rural areas. It also provides diverse financing channels for rural enterprises, helping them secure the funds needed to advance technological development. Rural technological innovation, in turn, directly contributes to enhanced economic resilience. By increasing agricultural productivity, optimizing resource allocation, and promoting industrial upgrading, innovation strengthens the rural economy’s ability to withstand shocks. Moreover, digital inclusive finance enhances the accessibility and convenience of financial services, which further supports technological innovation in rural areas, boosting economic resilience. With the backing of digital inclusive finance, rural innovative enterprises can more quickly develop and apply new technologies, improving productivity and market competitiveness, thereby reinforcing economic resilience. Additionally, technological innovation enhances human capital in rural areas by raising farmers’ technical skills and innovative capacity, further strengthening their resilience to economic risks.

H3: Digital inclusive finance enhances rural economic resilience by promoting rural technological innovation.

### Moderating effect

Digital infrastructure is a vital foundation for developing the digital economy and plays a key role in enhancing rural economic resilience. First, rural digital infrastructure serves as a prerequisite for the effective operation of digital inclusive finance. Components such as the internet, mobile communication networks, and data centers provide the essential technical support and service platforms required for digital financial services [30]. Well-developed digital infrastructure improves the coverage and transmission efficiency of financial services, enabling more rural residents and enterprises to access digital inclusive finance conveniently. Second, the construction of rural digital infrastructure directly contributes to strengthening rural economic resilience. By improving the efficiency and quality of information transmission, comprehensive digital infrastructure enhances transparency and market efficiency. Moreover, it facilitates the application of agricultural Internet of Things (IoT) technologies, enabling farmers to monitor and manage farmland in real time. This improves the precision and efficiency of agricultural production, boosting rural areas’ capacity for risk resistance and adaptability. Finally, robust digital infrastructure amplifies the positive effects of digital inclusive finance on rural economic resilience, allowing it to better strengthen risk resistance, adaptability, and innovation capacity. In regions with well-developed digital infrastructure, digital inclusive finance can deliver financial services more efficiently, helping rural enterprises and farmers manage market fluctuations and natural disasters more effectively. Conversely, in regions with weak digital infrastructure, the potential benefits of digital inclusive finance are limited.

H4: Rural digital infrastructure construction positively moderates the impact of digital inclusive finance on rural economic resilience.

### Variable measurement

Currently, there is no universally accepted standard for measuring economic resilience. Scholars primarily adopt single-indicator or composite-indicator approaches. While the single-indicator method avoids potential causality issues in variable selection, it oversimplifies the multidimensional nature of economic resilience. In contrast, the composite-indicator method captures multiple dimensions, offering a more comprehensive evaluation and is thus widely favored. Based on prior research [31], this study constructs a composite indicator system for rural economic resilience using 11 indicators across three dimensions: risk resistance, adaptive capacity, and innovation and transformation capacity.

Risk resistance reflects the capacity of rural economies to withstand shocks and maintain stability. This dimension is measured using four indicators. The total output value of agriculture, forestry, animal husbandry, and fisheries serves as an indicator of economic strength, where higher values signify stronger risk resistance. Total grain output measures the stability of agricultural production, with consistent yields demonstrating resilience to natural disasters. The ratio of irrigated area to arable land highlights the importance of irrigation infrastructure in supporting productivity, while the usage of agricultural fertilizers contributes to higher yields, further enhancing economic stability.

Adaptive capacity assesses how effectively rural economies can recover and adapt to changing conditions. Four indicators are used to evaluate this dimension. The per capita consumption level of rural residents indicates adaptability, with higher levels reflecting greater economic resilience. Engel’s coefficient for rural households measures consumption structure, where a lower coefficient signals more diversified spending and a stronger ability to adapt. Per capita disposable income is another key factor, as higher income levels provide greater economic strength and risk resistance. Additionally, completed investment in fixed assets by rural households demonstrates the potential for structural adjustments in agriculture, fostering economic growth and adaptability.

Innovation and transformation capacity captures the ability of rural economies to pursue innovation and develop new growth pathways in response to challenges. This dimension includes three indicators. The proportion of the rural population aged 15–64 reflects the demographic composition, with a higher share of younger and more productive individuals promoting innovation and receptiveness to technological advancements. The ratio of total agricultural machinery power to arable land serves as a measure of mechanization, where higher levels of mechanization enhance productivity and support economic transformation. Finally, the number of R&D personnel indicates the reserve of innovative talent, which is crucial for advancing agricultural production and strengthening the capacity for transformation.

This study applies the entropy method to determine indicator weights objectively, avoiding biases associated with subjective evaluations. The entropy method is widely used in similar research and allows for data-driven weight assignments (see Table 1 for results). Using this method and the composite indicator system, the study calculates a comprehensive rural economic resilience index for 30 provinces, municipalities, and autonomous regions in China (excluding Tibet, Hong Kong, Macau, and Taiwan) from 2013 to 2023.

**Table 1:** Comprehensive indicator system for rural economic resilience.

Primary Indicator	Secondary Indicator	Unit	Weight	Direction
Risk resistance	Total output value of agriculture, forestry, animal husbandry, and fisheries	100 million yuan	0.0696	+
	Total grain output	10,000 tons	0.1040	+
	Irrigated area/ Arable land area	%	0.1094	+
	Usage of agricultural fertilizers	10,000 tons	0.0772	+
Adaptive capacity	Engel’s coefficient for rural households	%	0.0914	–
	Per capita consumption level of rural residents	yuan/person	0.1301	+
	Per capita disposable income of rural residents	yuan/person	0.0632	+
	Completed investment in fixed assets by rural households	100 million yuan	0.1263	+
Innovative transformation capacity	Proportion of rural population aged 15–64 to total rural population	%	0.0337	+
	Ratio of total agricultural machinery power to arable land area	10,000 watts/hectare	0.1757	+
	Number of R&D personnel	persons	0.0194	+

## Methodology and data

### Variable design

#### Dependent variable.

The dependent variable is rural economic resilience $res_{i,t}$ Based on the previously constructed comprehensive indicator system, this paper uses risk resistance $res\_1_{i,t}$ adaptive capacity $res\_2_{i,t}$ and innovative transformation capacity $res\_3_{i,t}$ as dependent variables for empirical testing.

#### Core independent.

The core independent variable is digital inclusive finance $index_{i,t}$ which is measured using the digital inclusive finance index developed by the Institute of Digital Finance Peking University. For the purposes of analysis, this index is divided by 100. The index is a multidimensional, composite measure designed to assess the extent of digital inclusive finance development, focusing on the accessibility and reach of digital financial services in meeting people’s financial needs. In addition, this study examines the heterogeneity of the impact of digital inclusive finance on rural economic resilience from three dimensions: coverage breadth df_bre usage depth df_dep and degree of digitalization df_lev The df_bre primarily evaluates the extent of Alipay account penetration and the degree of bank card binding, reflecting the widespread adoption of digital financial services. The df_dep includes six service dimensions—payment, credit, insurance, investment, money market funds, and credit—which collectively capture the usage patterns of digital financial services. Finally, the df_lev is assessed across four dimensions: creditization, convenience, affordability, and mobility, which collectively evaluate the degree of digitalization within financial services [32].

#### Control variables.

To account for other factors that may affect rural economic resilience, this paper draws on relevant literature and selects five variables based on the research indicators and available data [33–35].

1. Human capital (lnedu): Human capital is an endogenous driver of rural economic resilience. This paper uses the average years of education for individuals aged six and above to represent human capital.
2. Agricultural loans (lnloan): Loans provided by financial institutions for agriculture are specifically aimed at farmers and help address the funding shortages encountered in their production and operations. This paper measures agricultural loans by their total amount.
3. Government agricultural support (lngov): Fiscal spending by the government plays an important role in agricultural development, although excessive long-term intervention may disrupt the internal regulatory mechanisms of agricultural development. This paper uses the total amount of agricultural fiscal expenditure to measure this factor.
4. Industrial structure (lnstruc): Adjustments in industrial structure contribute to the integration of rural industries and optimization of resource allocation. This paper measures industrial structure using the weighted average of industry share proportions.
5. Urbanization level (urb): The urbanization process drives the transfer of surplus labor to cities. This paper measures urbanization level by the proportion of the urban population to the total population in each province.

#### Mechanism variables.

To examine the mechanisms through which digital inclusive finance affects rural economic resilience, this study employs rural road network density (road) as an indicator of rural transportation infrastructure [36]. Given that railways and highways are the primary modes of transportation in China, this paper measures rural road network density by the “kilometer of transportation routes per square kilometer (including both highway and railway operating mileage).” The research focuses on 31 provinces and municipalities in China. Due to data availability limitations, data on agricultural R&D investment and personnel could not be obtained, making it impossible to assess rural technological innovation through input and efficiency indicators. Instead, this study employs an output-based measure of technological innovation, specifically the total number of patents granted in the agricultural sector (including agriculture, forestry, animal husbandry, and fishery), to represent the level of rural technological innovation (tech) [37]. A detailed explanation and definition of the relevant variables are provided in Table 2.

**Table 2:** Explanation of key variables.

Variable name	Symbol	Explanation	Mean	Standard deviation
Rural economic resilience	res	Index of rural economic resilience	0.277	0.124
Digital inclusive finance	index	Digital inclusive finance index	2.315	1.033
Human capital	lnedu	Average years of education per rural resident	2.055	0.0794
Financial support	lnloan	Balance of agricultural loans	8.784	0.874
Government agricultural support	lngov	Local fiscal expenditure on agriculture, forestry, and water affairs	5.709	0.500
Industrial structure	lnstruc	Weighted average of industry proportion (primary*1 + *s*econdary*2 + tertiary*3)	5.471	0.0525
Urbanization level	urb	Urban population/ total population	0.596	0.121
Rural digital infrastructure	lnband	Number of rural broadband internet access users	7.685	1.121
Transportation infrastructure	road	Rural road network density	0.982	0.520
Rural technological innovation	tech	Number of authorized agricultural patents	0.291	0.306

#### Moderating variable.

This paper uses the number of rural broadband internet access users (lnband) to measure rural digital infrastructure construction, serving as the moderating variable.

### Data sources

The data for the dependent and control variables in this study were obtained from publicly accessible sources, including the *China Rural Statistical Yearbook*, *China Population and Employment Statistical Yearbook*, *China Agricultural Machinery Industry Yearbook*, and the EPS database. These datasets are publicly available and can be accessed through the respective government department databases. To address missing data, linear interpolation was employed to ensure the completeness of the dataset and the accuracy of the analysis. The data for the core explanatory variable were sourced from the Digital Inclusive Finance Index, jointly compiled by the Institute of Digital Finance Peking University and Ant Financial Group. This index is also publicly accessible, and researchers can obtain the relevant data through the website of the Institute of Digital Finance at Peking University and its associated reports. To mitigate the influence of price fluctuations, the study uses the GDP deflator with 2012 as the base year to adjust price-related variables. Additionally, logarithmic transformations were applied to the relevant variables to ensure data smoothness and consistency.

### Model construction

#### Baseline regression model.

Based on panel data from 30 provinces, municipalities, and autonomous regions in China from 2013 to 2023, this paper employs a two-way fixed effects model for empirical testing at both the province and time levels. The specific model is as follows:


$$res_{i,t}=\alpha_{0}+\alpha_{1}index_{i,t}+\alpha_{2}control_{i,t}+\mu_{i}+\lambda_{i}+{�}_{i,t}$$ (1)

Where $res_{i,t}$ represents the rural economic resilience of province *t* in year *t*; $index_{i,t}$ represents the digital inclusive finance index of province *i* in year *t*; $control_{i,t}$ denotes the control variables; $\mu_{i}$ denotes individual fixed effects; $\lambda_{i}$ denotes time fixed effects; and ${�}_{i,t}$ is the clustered robust standard error. The parameter $\alpha_{1}$ is the core estimated parameter of this paper, used to measure the effect of digital inclusive finance on rural economic resilience.

To further explore the specific impact of digital inclusive finance on the sub-indices of rural economic resilience, this paper builds a regression model based on equation (1) for the sub-indices of rural economic resilience ($res\_n_{i,t}$), as follows:


$$res\_n_{i,t}=\alpha_{0}+\alpha_{1}index_{i,t}+\alpha_{2}control_{i,t}+\mu_{i}+\lambda_{i}+{�}_{i,t}$$ (2)

### Mediation effect model

This paper further explores the specific pathways through which digital inclusive finance impacts rural economic resilience. First, based on the theoretical mechanism analysis presented earlier, this paper examines the impact mechanism of digital inclusive finance on rural economic resilience through two pathways: transportation infrastructure and rural technological innovation. Second, in selecting the method for analyzing the impact mechanism, this paper draws on the approaches of Jiang (2022) [38] and Wu (2022) [39], conducting regression analysis on the core independent variable and the mechanism variables. Therefore, the regression model is set as follows:


$$M_{i,t}=\alpha_{0}+\alpha_{1}index_{i,t}+\alpha_{2}control_{i,t}+\mu_{i}+\lambda_{i}+{�}_{i,t}$$ (3)

Where $M_{i,t}$ represents the mechanism variable, used to test the relationship between digital inclusive finance and the mechanism variable. If $\alpha_{1}$ is significant and there is sufficient literature supporting that the mechanism variable can enhance rural economic resilience, it suggests that the mechanism variable is a pathway through which digital inclusive finance affects rural economic resilience.

### Moderation effect model

To examine the impact of rural digital infrastructure on the effect of digital inclusive finance on rural economic resilience, this paper draws on the study by Fang et al. (2022) [40] and establishes the following moderation effect model:


$$res_{i,t}=\alpha_{0}+\alpha_{1}index_{i,t}+\alpha_{2}lnband_{i,t}+\alpha_{3}control_{i,t}+\mu_{i}+\lambda_{i}+{�}_{i,t}$$ (4)

$$res_{i,t}=\alpha_{0}+\alpha_{1}index_{i,t}+\alpha_{2}lnband_{i,t}+\alpha_{3}index_{i,t}^{c}\times lnband_{i,t}^{c}+\alpha_{4}control_{i,t}+\mu_{i}+\lambda_{i}+{�}_{i,t}$$ (5)

Where $lnband_{i,t}$ represents the level of digital infrastructure construction in rural areas. For better model comparison and coefficient interpretation, the variables for digital inclusive finance and rural digital infrastructure are mean-centered, defined as $index_{i,t}^{c}=index_{i,t}-\bar{index}$ and $lnband_{i,t}^{c}=lnband_{i,t}-\bar{lnband}$. Equation (4) tests the impact of digital infrastructure on rural economic resilience. Equation (5) adds the interaction term ($index_{i,t}^{c}\times lnband_{i,t}^{c}$) to investigate the moderating role of digital infrastructure in the effect of digital inclusive finance on rural economic resilience. If both coefficients $\alpha_{2}$ and $\alpha_{3}$ are significant, it indicates that digital infrastructure moderates the relationship between digital inclusive finance and rural economic resilience. If they have the same sign, digital infrastructure strengthens the impact of digital inclusive finance on rural economic resilience; if they have opposite signs, digital infrastructure weakens the impact of digital inclusive finance on rural economic resilience.

#### Multicollinearity test.

To further ensure the accuracy and reliability of the empirical results, this study conducts a multicollinearity test on the core independent variable and its three sub-variables using the Variance Inflation Factor (VIF). According to the test results presented in Table 3, the VIF values for both the core explanatory variables and control variables are all below 5, indicating that there are no severe multicollinearity issues among the variables. Therefore, subsequent regression analysis can proceed.

**Table 3:** VIF analysis results.

Variable	index	df_bre	df_dep	df_lev
urb	4.88	4.88	4.87	4.94
lnstruc	4.68	4.74	4.58	4.32
lnloan	3.69	3.70	3.71	3.25
lngov	3.36	3.40	3.32	3.22
lnedu	1.83	1.83	1.84	1.81
index	2.22			
df_bre		2.28		
df_dep			2.36	
df_lev				1.50
MeanVIF	3.44	3.47	3.45	3.17

## Empirical results and analysis

### Baseline regression results analysis

Before conducting empirical analysis, this paper first performed F-tests, LR tests, and Hausman tests, all of which indicated that a fixed effects model should be used for regression analysis. To ensure the independence of explanatory variables, control variables were added to the regression one by one. The results show that the adjusted $R^{2}$ gradually increases, indicating the feasibility of this approach.

Table 4 reports the baseline regression results of the impact of digital inclusive finance on rural economic resilience. Columns (1) to (6) show that the regression coefficients of the core explanatory variable are significant at the 1% level. Column (6), which includes all control variables, estimates a marginal effect of 0.1041 for digital inclusive finance, meaning that each unit increase in digital inclusive finance raises rural economic resilience by 0.1041 units. This indicates that the development level of digital inclusive finance significantly promotes rural economic resilience in China, thereby verifying H1.

**Table 4:** Baseline regression results^1^.

Variable	(1) res	(2) res	(3) res	(4) res	(5) res	(6) res
index	0.1160^***^ (0.0353)	0.1136^***^ (0.0346)	0.1143^***^ (0.0299)	0.1110^***^ (0.0258)	0.1117^***^ (0.0253)	0.1041^***^ (0.0278)
lnedu		0.1405^**^ (0.0617)	0.1651^**^ (0.0642)	0.1722^**^ (0.0647)	0.1655^**^ (0.0649)	0.1627^**^ (0.0631)
lnloan			0.0292^*^ (0.0148)	0.0304^**^ (0.0138)	0.0316^**^ (0.0143)	0.0396^*^ (0.0231)
lngov				-0.0428^**^ (0.0162)	-0.0423^**^ (0.0163)	-0.0450^**^ (0.0170)
lnstruc					-0.1021 (0.1587)	-0.0922 (0.1546)
urb						-0.1135 (0.2154)
constant	0.1735^***^ (0.0161)	-0.1104 (0.1282)	-0.3986^**^ (0.1908)	-0.1844 (0.2006)	-0.1840 (0.1992)	0.3368 (0.8514)
$R^{2}$	0.7908	0.7949	0.8028	0.8135	0.8135	0.8139

^1^(a) Province fixed effects: controlled; Year fixed effects: controlled; Observations: 330. (b)

*** ,

** , and

* indicate significance at the 1%, 5%, and 10% levels, respectively. (c) Values in parentheses represent clustered robust standard errors.

Table 5 presents the impact of digital inclusive finance on the three sub-indices of rural economic resilience. It is evident that digital inclusive finance has a significant positive effect on adaptability and innovation transformation capacity, indicating that it primarily enhances rural economic resilience by improving these two dimensions. However, the regression coefficient for risk resilience is not statistically significant, which may be attributed to several factors. First, the effects of risk shocks on rural economic systems are often sudden and unpredictable, typically occurring in the short term. Consequently, the impact of digital inclusive finance on risk resilience may take time to become evident, suggesting the presence of a potential time lag in its effectiveness. Second, rural economic resilience to risk is influenced not only by the widespread adoption and use of financial tools but also by other factors, such as agricultural infrastructure development and government policy support. These factors may not have been fully accounted for in the model, potentially leading to a weaker direct impact of digital inclusive finance on risk resilience. Furthermore, when facing external shocks such as natural disasters or market fluctuations, rural residents may prioritize traditional coping mechanisms—such as government assistance or social networks—over digital inclusive finance products. As a result, the impact of digital inclusive finance may be more pronounced in enhancing adaptability and innovation capacity, while its role in risk resilience is likely constrained by other contextual factors.

**Table 5:** Regression results for sub-indices of rural economic resilience^1^.

Variable	(1) res_1	(2) res_2	(3) res_3
index	0.0311 (0.0217)	0.0301*** (0.0045)	0.0429** (0.0196)
constant	-0.3290 (0.4872)	0.1898 (0.1316)	0.4759 (0.5304)
$R^{2}$	0.5964	0.9732	0.2465

^1^Province fixed effects: controlled; Year fixed effects: controlled; Observations: 330. Control variables: yes.

### Robustness checks

To enhance the robustness of the research results, this study employs five methods for robustness checks: replacing the dependent variable, excluding municipalities, winsorizing data, adding control variables, and using an alternative econometric model.

1. Replacing the dependent variable: In constructing the rural economic resilience system using a composite indicator method, subjective selection of indicators and the lack of consensus on weight assignments may lead to causal confusion and measurement inaccuracies. Given the representativeness and continuity of single indicators, this study measures rural economic resilience using the agricultural output based on the economic sensitivity index constructed by Martin (2012) [41]. The specific calculation formula is as follows:
  $$res_{i,t}=\frac{AO_{i,t}-AO_{i,t-1}}{AO_{national,t}-AO_{national,t-1}}$$ (6)
  where $AO_{i,t}$ and $AO_{i,t-1}$ represent the agricultural output of province *i* in years *t* and t−1, and $AO_{national,t}$ and $AO_{national,t-1}$ represent the national agricultural output in years *t* and t−1. The regression results using this single indicator for rural economic resilience are shown in Column (1) of Table 6.

**Table 6:** Robustness test results^1^.

Variable	(1) res	(2) res	(3) res	(4) res	(5) res
index	1.1167** (0.5398)	0.1431*** (0.0323)	0.1033*** (0.0290)	0.1046*** (0.0266)	0.105*** (0.0149)
lninsur				-0.0032 (0.0070)	
lnopen				0.0080 (0.0072)	
constant	-10.8137 (15.4004)	0.6934 (0.9448)	0.3732 (0.8403)	0.4985 (0.8052)	0.246 (0.5012)
Province FE	Controlled	Controlled	Controlled	Controlled	
Year FE	Controlled	Controlled	Controlled	Controlled	
*N*	330	286	330	330	330
$R^{2}$	0.4566	0.8306	0.8049	0.8152	

^1^Control variables: yes.
2. Excluding municipalities: Municipalities possess significant resource and policy advantages, and their levels of digital inclusive finance and agricultural economic activity differ from those of other provinces. Therefore, including municipalities in the regression sample may interfere with the accuracy of the results. This study excludes data from the four municipalities and re-estimates the regression, with the results shown in Column (2) of Table 6.
3. Winsorizing data: To avoid the influence of extreme data on the regression results, this study applies winsorizing to the sample. The regression results after winsorizing are shown in Column (3) of Table 6.
4. Adding control variables: Considering that rural economic resilience may also be influenced by agricultural insurance and the degree of openness, this study adds control variables such as agricultural insurance premium income (lninsur) and the ratio of foreign direct investment to GDP (lnopen) for each province. The regression results with these additional control variables are shown in Column (4) of Table 6.
5. Using an alternative econometric model: Since the range of rural economic resilience scores is between 0 and 1, this study employs a Tobit model as an alternative to the original model for robustness testing. The regression results using the Tobit model are shown in Column (5) of Table 6.

The core explanatory variable in each robustness test remains significantly positive, consistent with the baseline regression results, confirming that the conclusion that digital inclusive finance promotes rural economic resilience is robust.

### Endogeneity test

The improvement in rural economic resilience enhances farmers’ ability to cope with risks, increases the disposable income of rural residents, and expands spending on digital network services, further incentivizing farmers to adopt digital inclusive finance services. Therefore, rural economic resilience may inversely affect the development of digital inclusive finance, leading to endogeneity issues and rendering parameter estimates unreliable. To address this, the DWH test was conducted, with the results showing a p-value of 0.0000, which significantly rejects the null hypothesis of “all variables are exogenous,” indicating the presence of endogeneity.

To address the issue of endogeneity, this study employs the average distance from each province to Hangzhou (indis) as an instrumental variable for digital inclusive finance. Hangzhou, as the epicenter of China’s digital economy and e-commerce, is equipped with advanced digital inclusive finance infrastructure and financial technology innovations. Recognized nationwide as a pioneer and experimental zone for digital inclusive finance, the distance to Hangzhou can effectively capture the external technological diffusion effects on the development of digital inclusive finance across provinces. We hypothesize that while the distance to Hangzhou may influence the degree of digital inclusive finance development, it does not directly impact rural economic resilience, thereby satisfying the exclusion restriction for an instrumental variable.

Table 7 reports the first-stage regression results from the two-stage least squares (2SLS) instrumental variable approach. The findings reveal a negative correlation between the distance from Hangzhou and the level of digital inclusive finance development—provinces further from Hangzhou exhibit lower levels of development. Additional tests confirm that the instrumental variable passes both the weak instrument and exogeneity tests, validating its appropriateness. The second-stage regression results, also presented in Table 7, indicate that digital inclusive finance significantly enhances rural economic resilience, with coefficients surpassing those in the baseline regression. This result is consistent with Jiang (2017) [42], who argues that instrumental variables often amplify the effects, thus making the higher coefficients plausible.

**Table 7:** Endogeneity test results^1^.

Variable	First-stage regression index	Second-stage regression res
indis	-0.1583*** (0.013)	
index		0.1151** (0.046)
constant	-9.3965*** (1.482)	2.8666*** (1.025)
$R^{2}$	0.987	0.712

^1^Province fixed effects: controlled; Year fixed effects: controlled; Observations: 330; Control variables: yes.

### Mechanism testing

To verify the mechanism hypotheses proposed in the theoretical analysis, this paper regresses model (3) to test the mediating roles of transportation infrastructure and rural technological innovation in the impact of digital inclusive finance on rural economic resilience.

Columns (1) and (4) of Table 8 show that the regression coefficients of digital inclusive finance on transportation infrastructure and rural technological innovation are both significantly positive. To enhance the credibility of the results, this paper conducts robustness checks by excluding municipality samples and performing 1% winsorizing on the total sample. According to the regression results in Columns (2) and (3), as well as Columns (5) and (6) of Table 8, the coefficients of digital inclusive finance on transportation infrastructure and rural technological innovation remain significant, confirming the reliability of the results. H2 and H3 are thus supported.

**Table 8:** Mechanism regression results^1^.

Variable	(1) road	(2) road	(3) road_w	(4) tech	(5) tech	(6) tech_w
index	0.1547* (0.0799)	0.2048* (0.1133)	0.1584* (0.0794)	0.6862*** (0.1807)	1.1349*** (0.2135)	0.6600*** (0.1735)
constant	0.1735*** (0.0161)	-0.1104 (0.1282)	-0.3986** (0.1908)	-0.1844 (0.2006)	-0.1840 (0.1992)	0.3368 (0.8514)
*N*	330	286	330	330	3286	330
$R^{2}$	0.5619	0.6062	0.5785	0.6126	0.6808	0.6333

^1^Province fixed effects: controlled; Year fixed effects: controlled; Control variables: yes.

### Moderation effect test

This paper regresses models (4) and (5) to examine the moderating role of rural digital infrastructure construction in the impact of digital inclusive finance on rural economic resilience. Column (1) of Table 9 shows that when rural digital infrastructure is added to the baseline regression, the coefficients of both digital inclusive finance and rural digital infrastructure are significantly positive. Column (2) includes the interaction term of digital inclusive finance and rural digital infrastructure without centering the variables, and the results remain significant, indicating that rural digital infrastructure construction has a positive moderating effect. To avoid regression errors caused by multicollinearity, the independent variable and moderating variable are mean-centered. The regression results, shown in Column (3) of Table 9, indicate that the coefficient of digital inclusive finance remains significantly positive and is noticeably larger than the baseline regression coefficient. This finding demonstrates that the impact of digital inclusive finance on rural economic resilience is enhanced as the level of rural digital infrastructure development improves. In other words, the more comprehensive the rural digital infrastructure construction, the greater the amplification of digital inclusive finance’s positive effect on rural economic resilience. Thus, H4 is supported.

**Table 9:** Moderation effect regression results^1^.

Variable	(1) res	(2) res	(3) res
index	0.1277*** (0.0282)	0.1037*** (0.0286)	0.1384*** (0.0254)
lnband	0.0471*** (0.0125)	0.0354** (0.0149)	0.0354** (0.0149)
index×lnband		0.0045** (0.0017)	
$index^{c}\times lnband^{c}$			0.0045** (0.0017)
constant	0.4198 (0.8366)	0.8991 (0.8736)	0.8991 (0.8736)
$R^{2}$	0.8351	0.8440	0.8440

^1^Province fixed effects: controlled; Year fixed effects: controlled; Observations: 330; Control variables: yes.

### Heterogeneity analysis

Based on dimensions of digital inclusive finance: Digital inclusive finance encompasses three dimensions: coverage breadth, usage depth, and degree of digitalization. Each dimension may have a different impact on rural economic resilience. This study uses these three dimensions as explanatory variables and conducts regression analyses to examine their effects on rural economic resilience. Columns (1)–(3) of Table 10 show that the regression coefficients of all three dimensions are significantly positive, with the coefficient for coverage breadth being the highest. This indicates that coverage breadth has the strongest promoting effect on rural economic resilience.

**Table 10:** Heterogeneity analysis regression results^1^.

Variable	(1) res	(2) res	(3) res	(4) res	(5) res	(6) res	(7) res	(8) res
df_bre	0.0890* (0.0510)							
df_dep		0.0505*** (0.0146)						
df_lev			0.0372*** (0.0100)					
index				0.1446*** (0.0251)	-0.0181 (0.1032)	0.1153** (0.0392)	0.0852* (0.0404)	0.1252*** (0.0407)
constant	-0.0334 (0.8954)	0.3034 (0.8106)	-0.1707 (0.8172)	4.1253 (2.4371)	-0.5746 (1.2999)	-1.0419 (1.8776)	0.5213 (1.2579)	-0.6067 (1.0710)
*N*	330	330	330	121	88	121	154	176
$R^{2}$	0.7976	0.8051	0.8102	0.8548	0.7965	0.8484	0.8266	0.8349

^1^(a) Province fixed effects: controlled; Year fixed effects: controlled; Control variables: yes. (b) Agricultural provinces: Henan, Shandong, Sichuan, Hebei, Heilongjiang, Hunan, Inner Mongolia, Hubei, Anhui, Jiangsu, Jilin, Guangxi, Liaoning, Jiangxi (14 provinces in total). (c) Non-agricultural provinces: Beijing, Tianjin, Shanghai, Zhejiang, Fujian, Guangdong, Hainan, Shanxi, Chongqing, Guizhou, Yunnan, Shaanxi, Gansu, Qinghai, Ningxia, Xinjiang (16 provinces in total).

Based on geographic regions: The uneven economic development across regions results in different levels of digital inclusive finance development, which may influence rural economic resilience differently. This study divides the 30 provinces into three major regions—eastern, central, and western—for subgroup analysis. Columns (4)–(6) of Table 10 show that digital inclusive finance significantly enhances rural economic resilience in the eastern and western regions, while the regression coefficient for the central region is negative and insignificant. This may be due to the relatively low level and slow growth of digital inclusive finance in the central region, combined with the region’s natural advantage in agricultural resources, which already supports high rural economic resilience and leads to diminishing marginal utility from digital inclusive finance.

Based on agricultural scale: To verify the heterogeneity of digital inclusive finance’s impact on rural economic resilience across different scales of agricultural development, this study classifies the 30 provinces into agricultural and non-agricultural provinces based on the proportion of agriculture, forestry, animal husbandry, and fisheries in their GDP. Columns (7) and (8) of Table 10 show that digital inclusive finance has a significant positive effect on rural economic resilience in both types of provinces but has a stronger effect in non-agricultural provinces. This may be because agricultural provinces are more likely to develop large-scale farming and have more productive fixed assets as collateral for loans, whereas non-agricultural provinces have less large-scale production. Digital inclusive finance helps alleviate financing constraints, provides financial support, and strengthens rural economic resilience in non-agricultural provinces.

## Conclusion and policy recommendations

### Research conclusions

The impact of digital inclusive finance on rural economic resilience is not only pivotal for assessing the effectiveness of digital inclusive finance policies but also holds significant practical implications for ensuring the sustainable and stable development of rural economies. Drawing on sample data from 30 provinces, municipalities, and autonomous regions in China from 2013 to 2023, this study empirically examines the effect of digital inclusive finance on rural economic resilience through econometric regression methods. The research investigates the direction, dimensions, and mechanisms by which digital inclusive finance influences rural economic resilience. Additionally, it explores the moderating effect of rural digital infrastructure on this relationship. Furthermore, this paper examines the heterogeneous impact of digital inclusive finance on rural economic resilience, considering factors such as the development of digital inclusive finance, rural geographic location, and regional agricultural development levels.

The research findings are as follows:

1. Digital inclusive finance significantly enhances rural economic resilience. Specifically, it plays a crucial role in strengthening the adaptability and innovation transformation capabilities of rural economies, although its impact on risk resilience is not statistically significant.
2. Digital inclusive finance further strengthens rural economic resilience by improving rural transportation infrastructure and promoting technological innovation in rural areas.
3. The development of rural digital infrastructure amplifies the effect of digital inclusive finance on rural economic resilience. The more advanced the digital infrastructure within a region, the more pronounced the impact of digital inclusive finance in enhancing rural economic resilience.
4. Improvements in the coverage, usage depth, and digitalization level of digital inclusive finance all contribute to enhancing rural economic resilience, with coverage showing the most significant positive effect.
5. Further analysis reveals that the development of digital inclusive finance significantly improves rural economic resilience in China’s eastern and western regions, while its impact on the central region is not statistically significant. Additionally, the effect of digital inclusive finance is notably stronger in non-agricultural provinces compared to agricultural provinces.

### Policy recommendations

Based on the research findings, it is recommended that, overall, the Chinese government should continue to expand the coverage of digital inclusive finance, particularly in rural areas. By enhancing financial education and literacy among farmers, strengthening policy support, and refining the regulatory framework through nationwide initiatives, the government can further amplify the role of digital inclusive finance in enhancing rural economic resilience. However, significant disparities exist across regions in terms of economic development, infrastructure construction, and financial service penetration rates. Therefore, implementing region-specific differentiated policies will not only foster more balanced regional economic development but also ensure the sustainable growth of digital inclusive finance. Based on the geographical characteristics of each region, the following policy recommendations are proposed:

1. Eastern region: Deepening digital financial innovation and application. The eastern region has a relatively mature economic development, a well-established financial service system, and comprehensive digital infrastructure. The primary challenge in this region lies in how to promote the innovative application of digital finance, enhance the depth and breadth of services, and further expand the coverage of financial products. The key priorities for the development of digital inclusive finance in this region are as follows:
  - Support and encourage the development of fintech companies, fostering the application of technologies such as big data, artificial intelligence, and blockchain in digital inclusive finance. In particular, innovative financial products and services should continuously emerge in areas like digital payments, smart credit, and precision agriculture finance. For example, big data and AI could be utilized to assess farmers’ creditworthiness, design loan and insurance products tailored to rural characteristics, reduce loan risks, and improve the precision and efficiency of financial services.
  - Strengthen the integration of existing financial service platforms and promote data sharing and interconnectivity between different platforms. Specifically, through cross-platform cooperation, seamless integration of various financial services should be encouraged to enhance the overall efficiency of rural financial services. Additionally, digital platforms can expand the functionality of agricultural e-commerce, integrating rural finance with e-commerce, logistics, and other industries through a one-stop service platform, thereby promoting the modernization of the rural economy.
2. Central and western regions: Focusing on addressing infrastructure and financial service coverage issues. Rural economies in the central and western regions still face significant infrastructure lag, particularly in areas such as transportation, communication, and digital infrastructure. As a result, the promotion of digital inclusive finance in these regions faces numerous challenges, especially in terms of infrastructure development, digital technology adoption, and financial service coverage. In addressing these issues, policy should focus on the following directions:
  - Increase investment in rural transportation and digital infrastructure, especially in expanding network coverage. Enhancing internet access in rural areas and establishing widespread mobile payment and e-commerce platforms are fundamental to the development of digital inclusive finance. In this context, it is crucial to ensure the stability and security of rural networks, addressing potential technological and trust barriers in digital financial services.
  - The financial service network in the central and western regions is relatively underdeveloped, with financial institutions concentrated in certain areas, which causes difficulties for rural residents and small enterprises to access financial services. Therefore, the government could provide subsidies, tax incentives, and other measures to encourage financial institutions to expand their presence in these regions. Additionally, supporting local financial institutions to collaborate with large banks via online platforms could help jointly develop financial products suited to rural characteristics, such as microloans and rural insurance, thus improving the coverage and accessibility of financial services.
3. In addition to independently advancing the development of digital inclusive finance in the eastern and central-western regions, the central government should strengthen inter-regional cooperation and promote both horizontal policy coordination and vertical policy integration. The government can create a cross-regional policy framework that addresses the unique needs of different regions while promoting the development of digital inclusive finance. For instance, the eastern region could share advanced technological expertise, policy support, and financial backing with the central and western regions to help them overcome development bottlenecks. Through resource sharing and collaborative efforts, a comprehensive advancement of digital inclusive finance can be achieved.

### Research limitations and future directions

Despite providing valuable empirical analysis of the relationship between digital inclusive finance and rural economic resilience, this study has several limitations.

1. While this study utilized data from multiple public channels, some data remains unavailable, particularly at more granular geographic units such as towns or villages. This limitation may result in the omission of certain nuances and regional variations. Future research could enhance data collection efforts, especially at the grassroots level, to obtain more representative and detailed data. Additionally, the use of innovative methods, such as big data technologies or satellite remote sensing data, could help overcome challenges related to data access.
2. This study has not fully accounted for spatial spillover effects. Rural economic resilience is not only influenced by the local level of digital inclusive finance development but also by the economic, technological, and financial conditions of neighboring regions. Given the cross-regional diffusion effects of digital inclusive finance, future studies could incorporate spatial spillover effects into their models to explore the interregional interaction mechanisms. This would provide a deeper understanding of the regional disparities and their combined impact on economic resilience.
3. This study focuses solely on China, whose rural economy and the development of digital inclusive finance have specific characteristics. Other countries, especially developing ones, may face different challenges and opportunities in the implementation of digital inclusive finance and the enhancement of rural economic resilience. Future research could adopt cross-cultural or cross-national comparative approaches to explore how digital inclusive finance impacts rural economic resilience in different countries and cultural contexts. This would not only enrich theoretical research but also provide a broader range of international experiences for policy formulation.
